# Evaluation of Drug Utilization Patterns during Initial Treatment in the Emergency Room: A Retroprospective Pharmacoepidemiological Study

**DOI:** 10.5402/2011/261585

**Published:** 2011-12-25

**Authors:** Chakrapani Cheekavolu, Rama Mohan Pathapati, Kudagi Babasaheb Laxmansingh, Satish Kumar Saginela, Veera Prasad Makineedi, Siddalingappa, Amitabh Kumar

**Affiliations:** ^1^Department of Pharmacology, Narayana Medical College Hospital, Chinthareddypalem, Andhra Pradesh, Nellore 524002, India; ^2^Department of Clinical Pharmacology, Narayana Medical College Hospital, Chinthareddypalem, Andhra Pradesh, Nellore 524002, India; ^3^Department of Emergency Medicine, Narayana Medical College Hospital, Chinthareddypalem, Andhra Pradesh, Nellore 524002, India; ^4^Department of Hospital Administration, Narayana Medical College Hospital, Chinthareddypalem, Andhra Pradesh, Nellore 524002, India

## Abstract

*Background*. We assessed the prescribing trends, average number of drugs per prescription, and cost per prescription during the initial contact of the patient with the physician in emergency room. *Methods*. This retro-prospective study was conducted over a period of six months. Medical records of two hundred patients were reviewed for prescribing patterns. *Results*. 52 different types of drugs (996 drugs) were prescribed in total 200 prescriptions during the mean time spent in emergency room of 2.8 ± 1.4 hours. The average number of drugs per prescription was 4.2 ± 1.2. 95% of drugs were prescribed by trade name. Average drugs cost per prescription was 784 ± 134 rupees (17USD). *Conclusion*. Polypharmacy remains the main form of irrational prescribing. Prescribing patterns of drugs were knowledge based rather than WHO criteria for rational use of drugs.

## 1. Introduction

Prescription writing requires updated knowledge and skill. It reflects the clinical judgment and behavior of the physicians. Rational prescription utilizes updated knowledge and adheres to prescribing policies [1]. Irrational prescribing trends lead to unproductive and risky treatment; such a prescription manifests in either exacerbation/prolongation of illness or higher costs or both. Drug utilization study analyses the prescribing patterns and justifies the rational use of drugs. Physicians often face challenges in selecting, initiating, and individualizing appropriate drug therapy for patients in the emergency room (ER). For this purpose, we assessed the prescribing trends, average number of drugs per prescription, and cost per prescription during the initial contact of the patient in the ER.

## 2. Materials and Methods

This retro-prospective study, conducted between December 2009 to March 2010, evaluates the use of drugs across the different indications in the ER. The necessary permission was obtained from the concerned authorities for data collection. Data regarding the type of emergency, drug, dose, form, route, and outcomes were collected. From the collected data, prescribing patterns, average number of drugs per prescription, cost per prescription, and duration of stay in the ER were analyzed. The cost data of each drug was obtained from CIMS and Drug today.

## 3. Statistical Analysis

The data was entered in the Microsoft excel spreadsheet 2003. The statistical analysis was conducted by means of Sigma graph pad prism software, Version-4, USA. Descriptive statistics for continuous data was presented as Mean ± SD and categorical data as actual numbers and percentages.

## 4. Results

In this study prescription of two hundred patients admitted in the ER was analysed. Indications for admission were shown in Table 1. 996 drugs belonging to 52 categories were prescribed during the time spent in ER of 2.8 ± 1.4 hours. Analysis of prescribing indicators reveals that the average numbers of drugs per prescription were 4.2 ± 1.2 and the cost per prescription was 784 ± 134 rupees. Among the prescribed drugs, 95% of drugs were prescribed by trade name, 63.45% were from the essential drug list, 79.96% were injections, and 5.19% were fixed-drug combinations. 57.69% belongs to four categories that include antibiotics, analgesics, proton pump inhibitors (PPI), and antiemetics, and the remaining 42.31% belong to 48 categories. Antibiotics were prescribed in 21.78%, analgesics in 16.85%, 12.04% PPI and 7.02% antiemetics. Among the antibiotics ceftriaxone prescription occupied 55 (25%). Only pantoprazole and ondansetron were among the PPI and antiemetics. Out of two hundred patients, 180 (90%) patients were shifted to the concerned department for further management. Death was observed in 11 (5.5%), and nine (4.5%) patients were discharged against medical advice during the course of treatment (Table 2).

## 5. Discussion

Drug utilization in the in-patient setting can provide mechanisms to assess drug prescribing trends, efficiency, and cost effectiveness of hospital formularies. We showed a pattern of drug prescribing in our emergency room during the initial contact by the emergency physician across different situations. They reflect the clinical judgment of the clinicians and the prescribing behaviour of the physicians during the initial contact. However, these prescribing patterns of drugs were awareness based rather than WHO criteria for rational use of drugs [2] or evidence based.

The average number of drugs per prescription, which was shown to be an important index of the standard of prescribing in this study, was 4.2 ± 1.2, which was higher than, WHO recommended that average number of drug per prescription should be 2.0 [3]. It is possible that when the patient was ill and the diagnosis was not yet confirmed at the time of admission, empirical polypharmacy will be required. However, it is always preferable to keep the mean number of drugs per prescription as low as possible to reduce the cost of treatment and to minimize the adverse effects and drug interactions.

The majority (95%) of drugs was prescribed by trade name. Physician prefers to write brand names of drugs of repute rather than by generic names. Prescribing by brand name may be an evidence of vigorous promotional strategies by pharmaceutical companies. Physicians also opine that prescribing by generic name may result in the purchase of drugs of uncertain bioavailability due to lack of awareness about bioequivalence and regulatory that control generic drugs. Prescribing by generic name helps the hospital pharmacy to have a better control of inventory. This will also help the pharmacy to purchase the drugs on contract basis, as the number of brands will be less. It can also reduce the confusion among the pharmacists while dispensing. Use of generic names of prescription eliminates the chance of duplication of drug products and reduces the cost of the patient.

The antibiotic utilization rate was 21.78%. The main reason for such an empirical use of antibiotics within 24 hours of admission is either overestimation of the severity of illness. They are also under pressure from patients attendants, who believe that the prophylactic antibiotic use provides rapid relief of disease. However, an interesting observation pertaining to the selection of antibiotic combination for the prophylaxis was the use of ceftriaxone with amikacin in the majority of cases despite the awareness of similar gram negative coverage inherent in this combination. In a study [4] it was shown that no specific infection or disease was identified in which the addition of an aminoglycoside to a broad-spectrum beta-lactam antibiotic therapy provided an advantage. Moreover, the addition of the aminoglycoside may increase the risk of nephrotoxicity.

Pantoprazole and ondansetron were the only drugs among the class of PPI and antiemetic, respectively. Pantoprazole sodium is available for intravenous (IV) use. The most frequently mentioned explanation for prescribing PPI without an indication was “GI prophylaxis”. Physicians consider that certain patients without oral feeding [5] or who were receiving nonsteroidal anti-inflammatory drugs, aspirin, corticosteroids, and chemotherapy are at a high risk of developing stress ulcers. Considering ondansetron as a first-line agent for relief of nausea or vomiting may be due to its better safety and efficacy profile over others [6].

The average number of drugs/prescription is 4.2 ± 1.2 with a mean cost per prescriptions 784 ± 134 INR. The high average cost of the drugs at the ER was due to the type and severity of the illness that the patients come with. It is also possible that junior hospital staff ordered most of prescriptions before the consultant evaluates the patient. However, we did not completely evaluate the cost of other aspects of health care such as transport, investigations, stay in the hospital, and other intangible costs, which, if calculated, will provide us with a more realistic picture of the existing situation.

## 6. Conclusion

During the mean stay of 2.8 hours in emergency room antibiotics usage was higher than all other groups of drugs, which is followed by analgesics. Polypharmacy remains the main form of irrational prescribing. 95% of drugs were prescribed by brand names. Prescribing patterns of drugs were need based rather than WHO criteria for rational use of drugs. To provide optimal, low-cost, and effective medicines to the patients, it should be made mandatory for the prescribers to attend regular continuing medical education to update their knowledge on WHO criteria for rational use of drugs. Additionally, hospital authorities should take stringent measures to minimize the influence of pharmaceutical companies and their representatives on the drug prescription.

## Figures and Tables

**Table 1:** Indications, prescribing trends, prescription cost, and duration of emergency stay of patients during the first contact with physician.

Indication	*n*	%	Average no. of drugs/prescription	Average cost/prescription (INR)	Duration of stay in ER (Hours)
CVS	26	13.0	5.4 ± 1.2	893 ± 162	3.0 ± 1.9
Poisoning	34	17.0	4.0 ± 1.1	606 ± 161	2.8 ± 1.1
CNS	13	6.5	4.5 ± 1.0	1099 ± 130	2.9 ± 2.4
Metabolic	18	9	4.0 ± 1.2	280 ± 108	2.4 ± 0.9
Traumatic	53	26.4	3.4 ± 1.5	240 ± 136	3.2 ± 1.4
Infection	38	19.0	3.8 ± 1.0	356 ± 109	2.5 ± 1.9
Renal	9	4.5	4.2 ± 0.9	310 ± 10	3.0 ± 1.0
Respiratory	9	4.5	4.0 ± 2.6	319 ± 180	3.1 ± 1.0

Total	200	100	4.2 ± 1.2	784 ± 134	2.8 ± 1.4

**Table 2:** Antibiotic utilization patterns across various emergencies.

drugs (%)	Antibiotics	NSAIDS	Proton pump inhibitors	Antiemetic	Opioid analgesics	Cortico steroids
Cardiac	8.19	8.19	4.09	4.09	—	—
Poison	14.85	7.42	12.37	3.46	2.47	—
Metabolic	18.07	12.04	15.06	4.81	—	2.4
Trauma	19.7	15.76	14.77	7.38	19.21	—
Central Nervous	27.77	13.88	20.83	13.88	2.77	—
Infection	54.02	22.98	11.49	11.49	—	—
Respiratory	34.48	11.49	5.74	9.19	—	11.49
Renal	17.54	8.77	8.77	12.28	—	8.77

Total	21.78	12.24	12.04	7.02	4.61	1.9

## References

[1] Kishore J (2006). *National Health Programs of India*.

[2] WHO Model list of essential medicines 16th list. http://www.who.int/medicines/publications/essentialmedicines/en/.

[3] Sharif SI, Al-Shaqra M, Hajjar H, Shamout A, Wess L (2007). Patterns of drug prescribing in a Hospital in Dubai, United Arab Emirates. *Libyan Journal of Medicine*.

[4] Sinert R, Bright L (2008). Empiric antibiotic therapy for sepsis patients: monotherapy with *β*-lactam or *β*-lactam plus an aminoglycoside?. *Annals of Emergency Medicine*.

[5] Jung R, MacLaren R (2002). Proton-pump inhibitors for stress ulcer prophylaxis in critically III patients. *Annals of Pharmacotherapy*.

[6] Patanwala AE, Amini R, Hays DP, Rosen P (2010). Antiemetic therapy for nausea and vomiting in the emergency department. *Journal of Emergency Medicine*.

